# Optimization and qualification of a functional anti-drug antibody assay for HIV-1 bnAbs

**DOI:** 10.1016/j.jim.2020.112736

**Published:** 2020-04

**Authors:** Michael S. Seaman, Miroslawa Bilska, Fadi Ghantous, Amanda Eaton, Celia C. LaBranche, Kelli Greene, Hongmei Gao, Joshua A. Weiner, Margaret E. Ackerman, David A. Garber, Yvonne J. Rosenberg, Marcella Sarzotti-Kelsoe, David C. Montefiori

**Affiliations:** a Center for Virology and Vaccine Research, Beth Israel Deaconess Medical Center, Boston, MA, USA; b Department of Surgery, Duke University Medical Center, Durham, NC, USA; c Department of Immunology, Duke University Medical Center, Durham, NC, USA; d Department of Microbiology and Immunology, Geisel School of Medicine at Dartmouth, Hanover, NH, USA; e Division of HIV/AIDS Prevention, Centers for Disease Control and Prevention, Atlanta, GA, USA; f PlantVax Corporation, Rockville, MD, USA

**Keywords:** Anti-drug antibody, HIV-1, Broadly neutralizing antibodies

## Abstract

The recent identification of human monoclonal antibodies with broad and potent neutralizing activity against HIV-1 (bnAbs) has resulted in substantial efforts to develop these molecules for clinical use in the prevention and treatment of HIV-1 infection. As with any protein therapeutic drug product, it is imperative to have qualified assays that can accurately detect and quantify anti-drug antibodies (ADA) that may develop in patients receiving passive administration of HIV-1 bnAbs. Here, we have optimized and qualified a functional assay to assess the potential of ADA to inhibit the neutralizing function of HIV-1 bnAbs. Using a modified version of the validated TZM-bl HIV-1 neutralization assay, murine anti-idiotype antibodies were utilized to optimize and evaluate parameters of linearity, range, limit of detection, specificity, and precision for measuring inhibitory ADA activity against multiple HIV-1 bnAbs that are in clinical development. We further demonstrate the utility of this assay for detecting naturally occurring ADA responses in non-human primates receiving passive administration of human bnAbs. This functional assay format complements binding-antibody ADA strategies being developed for HIV-1 bnAbs, and when utilized together, will support a multi-tiered approach for ADA testing that is compliant with Good Clinical Laboratory Practice (GCLP) procedures and FDA guidance.

## 1 Introduction

Passive administration of potent and broadly neutralizing anti-HIV-1 monoclonal antibodies (bnAbs) is a promising strategy for the prevention and/or treatment of HIV-1 infection (Caskey et al., 2019). Pre-clinical studies in non-human primate (NHP) models have provided strong evidence that passive administration of neutralizing antibodies can confer sterilizing protection against high dose or repeated low dose mucosal SHIV challenges (Gautam et al., 2018; Gautam et al., 2016; Julg et al., 2017a; Julg et al., 2017b; Moldt et al., 2012; Pegu et al., 2014; Rudicell et al., 2014; Saunders et al., 2015; Shingai et al., 2013), and can result in a transient decline of plasma viremia, and in some cases extended control of viral replication in the setting of established infection (Julg et al., 2017c; Nishimura et al., 2017; Barouch et al., 2013). Several bnAbs targeting different epitopes on the HIV-1 Envelope (Env) protein are currently in clinical development and being administered to both healthy volunteers as well as HIV-1 infected participants either on or off antiretroviral therapy (ART) treatment (Ledgerwood et al., 2015; Lynch et al., 2015; Bar-On et al., 2018; Caskey et al., 2015; Caskey et al., 2017; Cohen et al., 2018; Scheid et al., 2016). The bnAb VRC01, which targets the CD4 binding site, is currently in phase 2b efficacy testing in high risk individuals in the Americas as well as Southern Africa (AMP HIV Prevention Study) [HVTN 704/HPTN 085 (ClinicalTrials.gov identifier NCT02716675) and HVTN 703/HPTN 081 (ClinicalTrials.gov identifier NCT02568215)] (Gilbert et al., 2017), and several additional bnAbs, bnAb combinations, and bi-specific antibodies are in early phase clinical testing (Caskey et al., 2019).

Though the HIV-1-specific bnAb sequences were derived from humans, the passive delivery of bnAbs still has potential to stimulate an immune response in recipients and induce anti-drug antibodies (ADA). While the development of ADA to therapeutic protein drug products may be a relatively common occurrence, the clinical effects of ADA responses are highly variable, and can range from no measurable effect to responses that may be harmful and impact patient safety (Song et al., 2016; Guidance for Industry, 2014). The development of ADA may also impair the mechanism of action and affect the pharmacokinetic profile of a clinical drug product. Thus, the ability to detect and quantitate the induction of ADA is critical for evaluating potential immune responses that may impact safety and efficacy. For HIV-1 bnAbs, information on the incidence of ADA induction and the consequence of such responses will be important for their clinical advancement.

The U.S. Food and Drug Administration (FDA) has provided recommendations and guidance to facilitate the development and validation of assays for assessing the immunogenicity of therapeutic protein products tested in human clinical trials (Guidance for Industry, 2014; Immunogenicity Testing of Therapeutic Protein Products - Developing and Validating Assays for Anti-Drug Antibody Detection, 2019). A multi-tiered testing approach for ADA is recommended in which samples from clinical trial participants are first screened in a binding antibody-based assay designed to detect low levels of low- and high-affinity ADA (Tier 1). Samples testing positive in the screening assay are then further evaluated in a competition-based confirmatory assay to define specificity (Tier 2). Samples identified as positive in the confirmatory tier of testing can be further characterized in titration assays and functional neutralization assays (Tier 3). In vitro ADA neutralization assays can evaluate the potential for ADA positive samples to inhibit the pharmacologic activity of the clinical drug product (Gupta et al., 2007; Wu et al., 2016). Such functional ADA assays are not used to confirm an ADA response, but rather are used to characterize the response to determine whether the ADA might result in a lack of pharmacologic benefit. Thus, the assay method utilized for assessing Tier 3 functional ADA potential should be based on the mechanism of action of the drug product. For HIV-1 bnAbs, the critical mechanism to assess for potential impairment by ADA is virus neutralizing activity.

Given the number of HIV-1 bnAbs currently in clinical testing, and many additional candidates in early stage development, there has been an increased emphasis on developing this multi-tiered strategy for measuring ADA against HIV-1 bnAb drug products. In this study, we developed a functional assay to enable the detection and quantitation of inhibitory Tier 3 level ADA against a panel of HIV-1 bnAbs that are in clinical development. Assay optimization and qualification utilized known anti-bnAb ADA to evaluate the parameters of linearity, range, limit of detection, specificity, and precision. All assays were performed in a laboratory compliant with Good Clinical Laboratory Practice (GCLP) guidelines (Sarzotti-Kelsoe et al., 2009).

## 2 Materials and methods

The method described in this report is a modification of the validated TZM-bl neutralization assay which has been previously published (Montefiori, 2009; Sarzotti-Kelsoe et al., 2014). The TZM-bl assay measures antibody-mediated neutralization of HIV-1 as a function of reduction in HIV-1 Tat-regulated luciferase reporter gene expression after a single round of infection by Env-pseudotyped viruses. Details regarding cell lines, preparation and titration of HIV-1 Env pseudotyped viruses, and optimized assay conditions can be found in Sarzotti-Kelsoe, et al. (Sarzotti-Kelsoe et al., 2014). A detailed protocol of the parental TZM-bl neutralization assay and other supporting material are available on the Los Alamos HIV Immunology Database (https://www.hiv.lanl.gov/content/nab-reference-strains/html/home.htm).

### 2.1 Monoclonal antibodies and serological reagents

Human broadly neutralizing monoclonal antibodies (mAbs) 10–1074 and 3BNC117 were provided by the laboratory of Dr. Michel Nussenzweig at The Rockefeller University (New York, NY). Anti-10-1074 and anti-3BNC117 mAbs were provided by the Duke Human Vaccine Institute using a mouse hybridoma cell line that was obtained from CellDex Therapeutics (Hampton, NJ). BnAbs PGT121 and PGDM1400, and mouse anti-PGT121 and anti-PGDM1400 mAbs were provided by the laboratory of Dr. Dan Barouch at Beth Israel Deaconess Medical Center (Boston, MA). BnAb VRC01 and mouse anti-VRC01 mAb were provided by Dr. John Mascola at the National Institutes of Health Vaccine Research Center. Normal human serum (NHS) samples were obtained from healthy donors with the approval of the Duke University Medical Center Institutional Review Board (IRB), and aliquots stored at -70^o^ C. Serum samples from NHP bnAb passive infusion studies were provided by Dr. Yvonne Rosenberg at PlantVax Inc. (Rockville, MD) and Dr. David Garber at Centers for Disease Control and Prevention (Atlanta, GA). All serum samples were heat-inactivated at 56^o^ C for 30 min prior to use.

### 2.2 General format for the TZM-bl ADA neutralization assay

A modification of the validated TZM-bl neutralization assay (Montefiori, 2009; Sarzotti-Kelsoe et al., 2014) was used to measure ADA as a function of reduced neutralizing activity of a target bnAb. Neutralization was quantified by Tat-regulated luciferase reporter gene expression after a single round of virus infection in the presence of a partial inhibitory dose of bnAb. Serological reagents to be tested for ADA activity were serially diluted in duplicate wells in 96-well flat-bottom culture plates containing growth media with a fixed concentration of target bnAb. Once the dilution steps were completed, Env-pseudotyped virus that was previously titrated for optimal infectivity was added, and the plates incubated for 1 h at 37^o^ C. Freshly trypsinized TZM-bl cells in growth media containing an optimal concentration of DEAE-dextran were then added to each well. One set of eight control wells received TZM-bl cells alone (background cell control), another set of eight control wells received cells plus virus (virus control), and another set of eight control wells received cells plus virus plus the fixed concentration of bnAb (bnAb neutralization control). Following a 48 h incubation at 37^o^ C, 150 μl culture medium was removed and 100 μl of Luciferase reporter gene assay system reagent was added. Cells were allowed to lyse for 2 min at room temperature, and 150 μl of cell lysate was transferred to 96-well black solid plates and luminescence read using a Victor 3 luminometer (Perkin-Elmer Life Sciences, Shelton CT). Percent ADA neutralization was determined for each sample dilution by calculating the difference in average relative light units (RLU) between virus control (cells + virus) and test wells (cells + sample + virus), dividing this result by the difference in average RLU between virus control (cells + virus) and bnAb neutralization control wells (cells + bnAb + virus), and multiplying by 100. ADA titers are expressed as the reciprocal sample dilution required to decrease the neutralizing activity of the bnAb by a desired percent (i.e. serum ID_50_ titers represent ADA activity that inhibits 50% of bnAb neutralization).

It should be noted that additional optimization experiments were performed to investigate whether increased sensitivity of ADA detection would be achieved by incubating the ADA test sample with the target bnAb for 1 h at 37 °C prior to the addition of Env-pseudovirus. No difference in ADA activity was observed in comparison assays with or without the 1 h incubation (data not shown), thus the final ADA assay protocol does not include this incubation step.

## 3 Results

### 3.1 Optimal range of bnAb neutralization levels

The strategy employed for quantifying functional anti-bnAb antibodies was based on a modification of the validated TZM-bl assay protocol for measuring neutralizing antibodies against HIV-1 (Montefiori, 2009; Sarzotti-Kelsoe et al., 2014). Thus, serial dilutions of test samples (e.g. patient serum or anti-idiotype mAbs) were assayed in the presence of a single fixed concentration of the bnAb of interest and an HIV-1 Env pseudovirus selected for known sensitivity to the bnAb. Results were expressed as the maximum percent reduction of bnAb neutralizing activity against the indicator virus, and the sample dilution at which there is a 50% reduction in bnAb neutralizing activity (ID_50_ titer). The presence of a fixed concentration of the target bnAb is the only modification of the TZM-bl assay for the purpose of measuring ADA and therefore was the critical parameter for optimization.

**Fig. 1:**
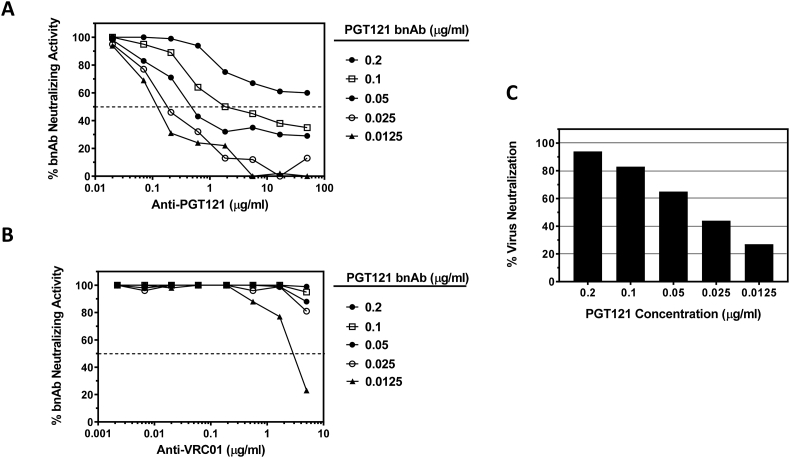
Optimization of anti-PGT121 detection in the TZM-bl assay. Serial dilutions of anti-PGT121 mAb (A) or anti-VRC01 mAb (B) were assayed in the presence of five different concentrations of bnAb PGT121 against virus RHPA4259. Data are graphed as the percent neutralizing activity of PGT121 at each dilution of ADA. (C) Percent neutralization of virus RHPA4259 observed at the five different concentrations of PGT121 in the absence of anti-idiotype antibody.

Optimization experiments were performed to determine an acceptable range of bnAb inhibition values that maximized sensitivity for detecting and quantifying functional ADA while minimizing non-specific effects. For these assays, murine monoclonal anti-idiotype antibodies for each bnAb were utilized as positive control ADA samples. Experiments testing serial dilutions of anti-PGT121 and anti-VRC01 mAbs in the presence of five different fixed concentrations of bnAbs PGT121 and VRC01 were performed. HIV-1 Env pseudovirus RHPA4259 was utilized as the indicator virus for measuring both PGT121 and VRC01 neutralizing activity, as this isolate is highly sensitive to both bnAbs. As shown in Fig. 1, anti-PGT121 mAb reduced the neutralizing activity of PGT121 in a dose-dependent manner. Furthermore, the potency of anti-PGT121 activity diminished as the fixed assay concentration of PGT121 bnAb was increased, demonstrating drug interference. Greatest sensitivity for anti-PGT121 detection was observed at the lowest fixed concentration of PGT121 tested (0.0125 μg/ml); however this concentration of PGT121 resulted in <30% neutralization potency against the indicator virus in the absence of ADA (Fig. 1C). Only at this lowest concentration of PGT121 bnAb was non-specific activity observed with the anti-VRC01 anti-idiotype antibody (Fig. 1B), indicating that lower thresholds of bnAb neutralizing activity may limit the specificity and precision of ADA detection. Fixed PGT121 concentrations of 0.025, 0.05 and 0.1 μg/ml resulted in a range of 44–83% neutralization of the indicator virus, and permitted detection of >50% reduction in PGT121 neutralizing activity by anti-PGT121 mAb concentrations >0.2 μg/ml.

**Fig. 2:**
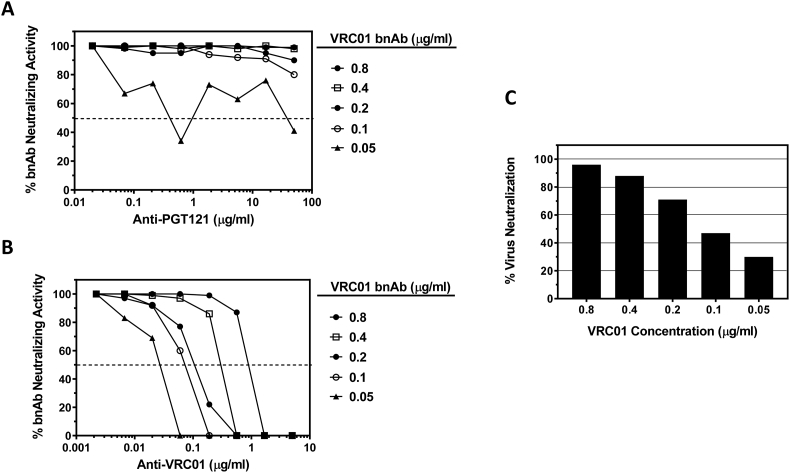
Optimization of anti-VRC01 detection in the TZM-bl assay. Serial dilutions of anti-PGT121 mAb (A) or anti-VRC01 mAb (B) were assayed in the presence of five different concentrations of bnAb VRC01 against virus RHPA4259. Data are graphed as the percent neutralizing activity of VRC01 at each dilution of ADA. (C) Percent neutralization of virus RHPA4259 observed at the five different concentrations of VRC01 in the absence of anti-idiotype antibody.

Similar results were obtained with VRC01 bnAb (Fig. 2). Anti-VRC01 mAb reduced the neutralizing activity of VRC01 in a dose-dependent manner, and the potency of functional ADA activity diminished as the fixed assay concentration of VRC01 bnAb was increased. Greatest sensitivity for anti-VRC01 detection was observed at the lowest fixed concentration of VRC01 tested (0.05 μg/ml; Fig. 2B); however this concentration of VRC01 resulted in <30% neutralization potency against indicator virus RHPA4259 in the absence of ADA (Fig. 2C). As observed with PGT121, only at this lowest fixed concentration of VRC01 bnAb was non-specific activity detected with the anti-PGT121 anti-idiotype antibody (Fig. 2A). Fixed VRC01 assay concentrations of 0.1, 0.2 and 0.4 μg/ml resulted in a range of 47–88% neutralization against the indicator virus and permitted detection of >50% reduction in VRC01 neutralizing activity by ADA concentrations >0.1 μg/ml.

Together these results highlight the impact of drug interference and the need to define an acceptable range of bnAb inhibition values that maximize sensitivity while minimizing non-specific effects. From these experiments we deduce that the assay is optimal when bnAb is present at a dose that results in 40–80% neutralization in the absence of ADA. Furthermore, these data demonstrate that optimal conditions can improve the precision of the assay to permit 50% reduction in neutralization as a reliable measure of functional ADA titer.

### 3.2 Limit of detection (LOD) and linearity

**Fig. 3:**
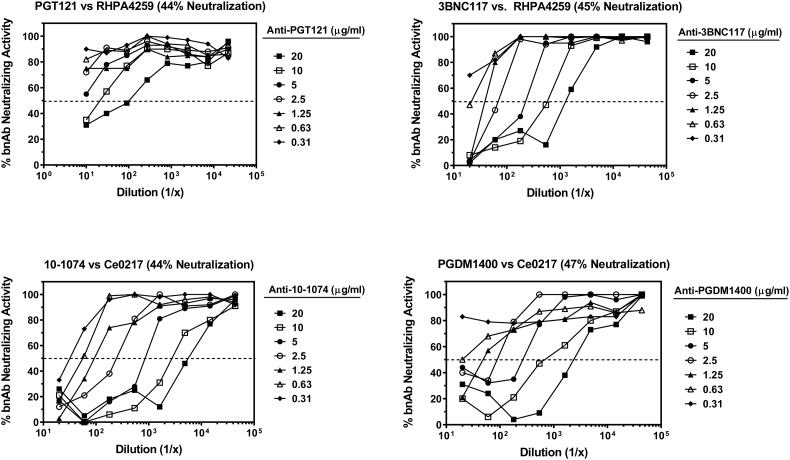
Limit of detection of ADA against bnAbs PGT121, 3BNC117, 10–1074, and PGDM1400. Anti-idiotype antibodies specific for each bnAb were diluted in PBS at seven different concentrations prior to titrating in the assay. Data are graphed as the percent neutralizing activity of the bnAb at each dilution of ADA. Percent inhibition of virus infectivity by each bnAb in the absence of anti-idiotype antibody is indicated above the corresponding figure.

The LOD of four different murine anti-idiotype antibodies (anti-PGT121, anti-3BNC117, anti-10-1074, and anti-PGDM1400) was measured by diluting each antibody in PBS at seven different concentrations (range 0.31–20 μg/ml) prior to titrating each sample in the assay. HIV-1 Env pseudoviruses RHPA4259 and Ce703010217_B6 (Ce0217) were used as indicator viruses for detecting the neutralizing activity of either PGT121 and 3BNC117, or 10–1074 and PGDM1400, respectively. Samples were assayed in the presence of a partial inhibitory dose of the corresponding bnAbs (PGT121, 0.05 μg/ml; 3BNC117, 0.04 μg/ml; 10–1074, 0.03 μg/ml; PGDM1400, 0.01 μg/ml). Positive functional ADA activity that reached a threshold of 50% reduction in respective bnAb neutralizing activity was observed for concentrations >5.0 μg/ml for anti-PGT121, >0.31 μg/ml for anti-3BNC117, as low as 0.31 μg/ml for anti-10-1074, and > 0.63 μg/ml for anti-PGDM1400 (Fig. 3). The linearity of the neutralization reduction curves varied depending on the ADA antibody and the fixed concentration of bnAb used. Under optimal conditions (40–80% virus neutralization by the bnAb), the neutralization reduction curves for anti-idiotype antibodies were generally linear between inhibition levels of 30% and 75%.

### 3.3 Specificity

**Fig. 4:**
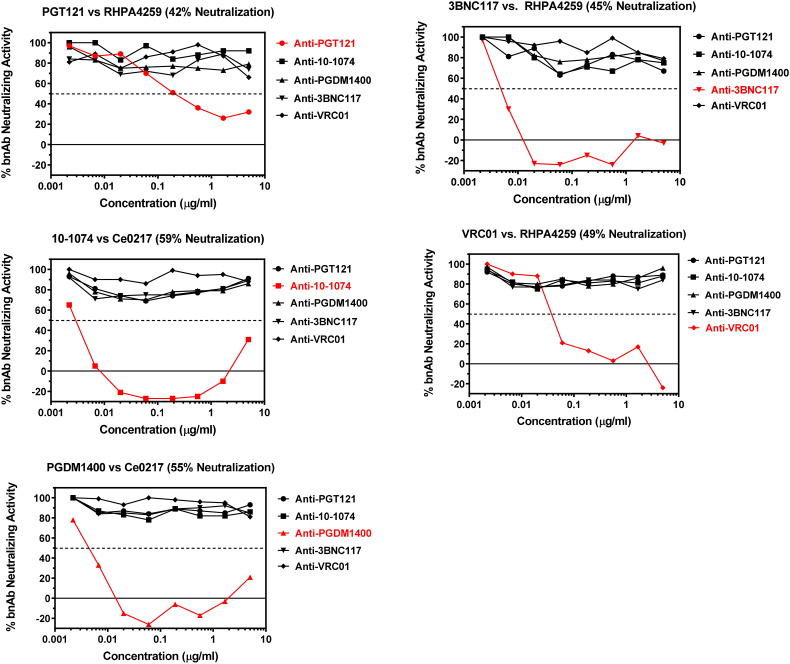
Specificity of anti-bnAb ADA antibodies. Serial dilutions of anti-idiotype antibodies against five different bnAbs were assayed in the presence of each bnAb and a sensitive strain of HIV Env pseudovirus. Virus/bnAb combination is shown above each corresponding figure. Percent neutralization by each bnAb in the absence of anti-idiotype antibody is also indicated. Matched bnAb/anti-idiotype combinations are highlighted in red. (For interpretation of the references to colour in this figure legend, the reader is referred to the web version of this article.)

Using the optimized conditions described above, we further tested the specificity of the assay. Specificity was determined by assaying five different murine anti-idiotype antibodies (anti-PGT121, anti-10-1074, anti-PGDM1400, anti-3BNC117 and anti-VRC01) in the presence of each bnAb. As shown in Fig. 4, positive activity (i.e. reduction in bnAb neutralizing activity) was observed only when the bnAb and anti-idiotype antibody were matched. Non-matched anti-idiotype antibodies were consistently negative in each case. We conclude that these murine anti-idiotype antibodies are highly specific for their matched bnAb. A 50% reduction in bnAb neutralizing activity was seen at 0.19 μg/ml for anti-PGT121, 0.003 μg/ml for anti-10-1074, 0.005 μg/ml for anti-PGDM1400, 0.005 μg/ml for anti-3BNC117, and 0.04 μg/ml for anti-VRC01. Neutralization potencies of the bnAbs against the indicator HIV-1 pseudoviruses in the absence of ADA were all within our pre-defined acceptance range of 40–80%.

**Fig. 5:**
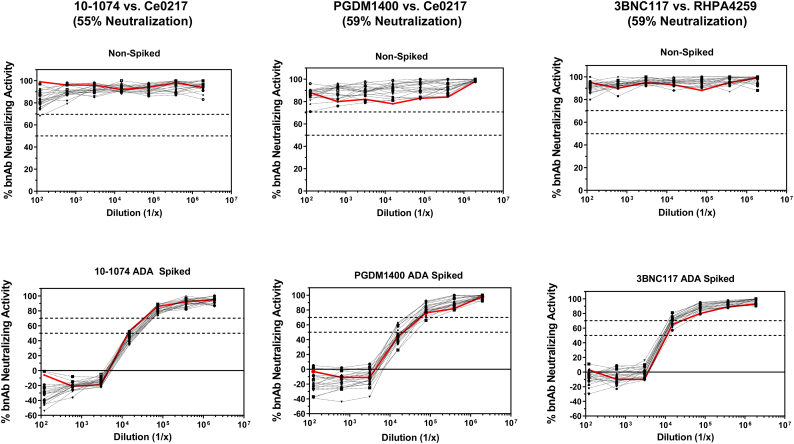
Effect of normal human serum on functional ADA detection. NHS samples (*n* = 20) were tested in functional ADA assays either alone or spiked with anti-idiotype antibodies at a concentration of 200 μg/ml and titrated in the presence of the corresponding bnAb. Titrations of PBS control with or without spiked anti-idiotype antibodies are highlighted red. Virus/bnAb combination is shown above corresponding figures with percent neutralization in the absence of anti-idiotype antibodies. Dashed lines represent 50% or 30% inhibition levels of bnAb neutralizing activity (note that 30% inhibition equals 70% bnAb neutralizing activity in the figures). (For interpretation of the references to colour in this figure legend, the reader is referred to the web version of this article.)

The specificity experiments described above used ADA diluted in PBS prior to assay. Because clinical specimens will be human serum samples, we investigated the impact of murine anti-idiotype antibodies spiked into human serum samples from healthy HIV-1 naive individuals. Experiments were performed with twenty NHS samples spiked with either anti-10-1074, anti-PGDM1400 or anti-3BNC117 antibody at 200 μg/ml and titrated in the presence of a partial inhibitory dose of the corresponding bnAbs (10–1074, 0.05 μg/ml; PGDM1400, 0.025 μg/ml; 3BNC117, 0.06 μg/ml). NHS samples that were not spiked with ADA served as negative controls to assess non-specific background activity in the optimized functional ADA assay. For comparison, we additionally assayed PBS with or without spiked ADA. As shown in Fig. 5, neutralization curves were similar among the twenty spiked NHS on a per ADA basis, and were comparable to ADA-spiked PBS control samples. We conclude that NHS did not affect the sensitivity of functional anti-drug antibodies. Furthermore, titrations of non-ADA spiked NHS sample exhibited minimal levels of background inhibition in the presence of each bnAb. We utilized these results to calculate a statistical threshold for positivity using the recommended cut-off of 3.09 standard deviations from the mean of background inhibitory activity (Shankar et al., 2008). Given that positive ADA thresholds of 29, 17, and 26% inhibition were calculated as positive cut-offs for PGDM1400, 3BNC117, and 10–1074 bnAbs respectively, we conclude that 30% inhibition of bnAb activity (i.e serum ID_30_ titer) may be a reliable measure for determining a positive functional ADA response. However, our data further demonstrated that measured ADA ID_30_ titers were consistently more variable across the 20 spiked NHS when compared to ADA ID_50_ titers, with the standard deviation in ID_30_ titers being 2 to 5-fold higher across the three different bnAbs. This is likely due to 50% neutralization being on a more linear portion of the ADA inhibition curve and thus less variable (for example the 3BNC117 ADA spiked neutralization curve). Thus, while we demonstrate that 30% inhibition may be a statistically relevant cut-off for positivity, we recommend 50% inhibition as a more stringent and reliable measure of functional ADA titer.

### 3.4 Precision

**Fig. 6:**
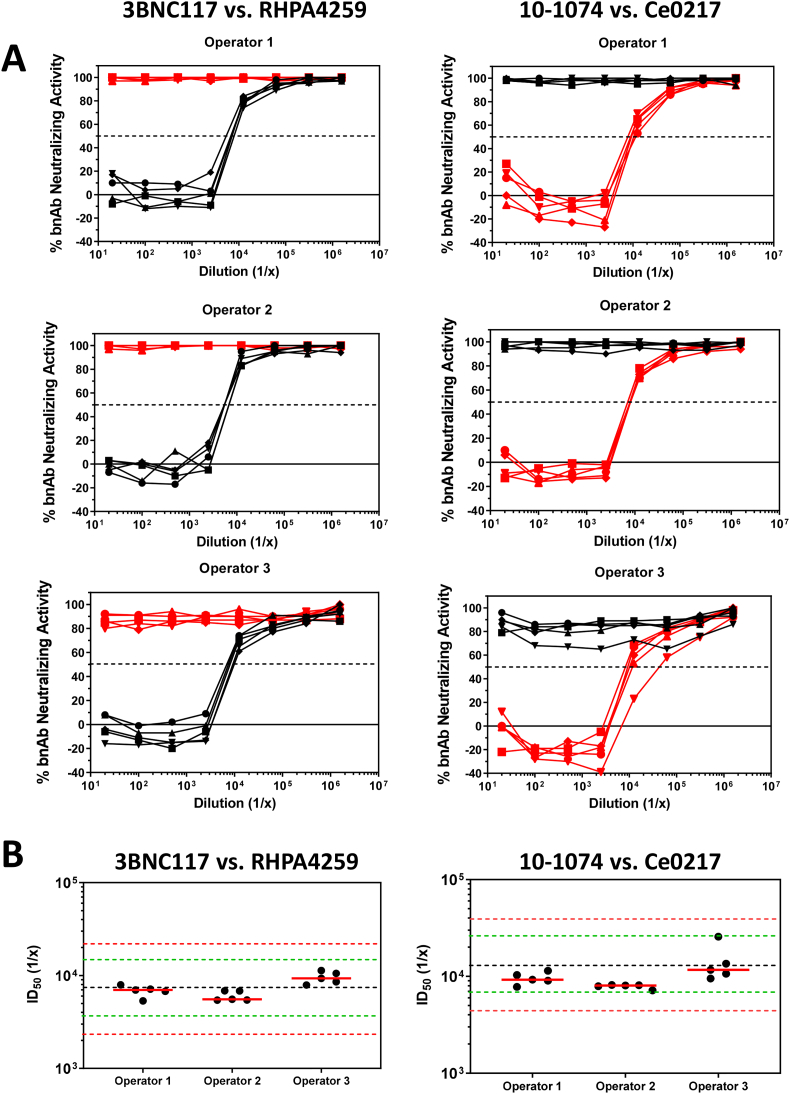
Intra- and inter-operator variability of the TZM-bl functional ADA assay. (A) Three operators assayed serial dilutions of NHS spiked with either anti-3BNC117 (black lines) or anti-10-1074 (red lines) mAbs five times each in the presence of either 3BNC117 or 10–1074 bnAbs. Virus/bnAb combination is shown above corresponding figures, and percent neutralizing activity in the absence of anti-idiotype antibodies was within the acceptance range of 40–80% for all assays. (B) The mean titers for each individual operator are indicated as solid red bars for the corresponding bnAb as indicated. The black dashed line is the mean of all tests. Green dashed lines correspond to a 2-fold range above and below the mean. Red dashed lines correspond to a 3-fold range above and below the mean. (For interpretation of the references to colour in this figure legend, the reader is referred to the web version of this article.)

Intra- and inter-operator variability was tested by having three operators perform a set of assays five times on different days spanning three weeks. Each operator tested anti-3BNC117 and anti-10-1074 mAbs in the presence of either 3BNC117 or 10–1074 bnAb in each set of assays. All anti-idiotype antibodies were spiked in NHS and tested in the presence of both bnAbs. Consistent with our specificity findings described above, no operator in any test detected ADA activity when anti-3BNC117 was tested in the presence of 10–1074, or when anti-10-1074 was tested in the presence of 3BNC117 (Fig. 6A). Positive ADA activity was only detected with anti-idiotype antibodies that were assayed against the matched bnAbs. Results of the matched anti-idiotype/bnAb combinations are shown in Fig. 6B. For both anti-3BNC117 and anti-10-1074 measurements, 100% of individual serum ID_50_ titers were within 2-fold of the mean, and the coefficient of variation for all intra- and inter-operator runs was 25% and 43%, respectively. These results demonstrate a high level of precision of the assay.

### 3.5 Detection of induced ADA responses in rhesus macaques

As another test of precision, we utilized the optimized functional ADA assay format described above to screen serum samples from rhesus macaques infused with different human bnAbs. Rhesus macaques often mount an ADA response to many human antibodies (Rosenberg et al., 2015), thus providing an opportunity to assess the ability of the optimized assay to detect and quantitate a naturally induced ADA response. In this experiment, monkeys were infused either once or twice with one of three different antibodies: PGT121-YTE, 3BNC117-YTE, or PGDM1400-YTE. Precision and specificity were assessed by testing longitudinal serum samples from each monkey in optimized assays for measuring functional ADA activity against bnAb PGT121 and PGT121-YTE. Unlike the non-immunogenic PGT121, 4/8 macaques generated ADA responses that interfered with PGT121 neutralizing activity at low levels (peak serum ADA ID_50_ titers ranging from 144 to 660) following a single infusion of PGT12-YTE. Dramatic increases in functional ADA potency (peak ID_50_ titers of 1,183 and 17,579) were observed in the two animals that received a second infusion (Table 1). No ADA was detected in these macaques at baseline, prior to infusion, and animals injected with YTE mutants of 3BNC117 and PGDM1400 showed no anti-PGT121 inhibitory ADA activity. A second assay performed by the same operator 2 weeks later using PGT121-YTE indicated that the two sets of results agreed within 3-fold of each other and 9/10 positive results agreed within 2-fold. These data demonstrate an acceptable level of assay precision and specificity using natural samples of a genuine ADA response to a HIV-1 bnAb.

**Table 1:** ADA detection in nonhuman primates. Rhesus macaques were infused with either PGT121-YTE, 3BNC117-YTE or, PGDM1400-YTE and assayed for ADA activity against PGT121 (original) and PGT121-YTE (repeat). Time points are days post 1st or 2nd infusion. Data from two independent assays are shown.

mAb injected	Monkey ID	Time point	ADA ID50 in TZM-bl cells	
			RHPA4259.7	RHPA4259.7
			Anti-PGT121, original	Anti-PGT121-YTE, repeat
3BNC117-YTE	T765	1st day0	<20	<20
		1st day14	<20	<20
		2nd day0	<20	<20
		2nd day14	<20	<20
3BNC117-YTE	T766	1st day0	<20	<20
		1st day14	<20	<20
		2nd day0	<20	<20
		2nd day14	<20	<20
PGDM1400-YTE	T767	1st day0	<20	<20
		1st day21	<20	<20
		2nd day0	<20	<20
		2nd day7	<20	<20
		2nd day20	<20	<20
PGDM1400-YTE	T768	1st day0	<20	<20
		1st day21	<20	<20
		2nd day0	<20	<20
		2nd day7	<20	<20
		2nd day20	<20	<20
PGT121-YTE	T769	1st day0	<20	<20
		1st day14	**144**	**140**
		1st day21	**660**	**555**
		2nd day0	**361**	**298**
		2nd day7	**17,579**	**10,851**
		2nd day20	**8,768**	**5,822**
PGT121-YTE	T770	1st day0	<20	<20
		1st day21	<20	<20
		2nd day0	**125**	**103**
		2nd day7	**1,183**	**751**
		2nd day20	**645**	**538**
PGT121-YTE	12D101	Day 0	<20	<20
		Day 7	<20	<20
		Day 16	<20	<20
	11D042	Day 0	<20	<20
		Day 7	<20	<20
		Day 16	**156**	**70**
	12D046	Day 0	<20	<20
		Day 7	<20	<20
		Day 16	**171**	**304**
	09D181	Day 0	<20	<20
		Day 7	<20	<20
		Day 16	<20	<20
PGT121-YTE	13D036	Day 0	<20	<20
		Day 4	<20	<20
		Day 8	<20	<20
	13D077	Day 0	<20	<20
		Day 4	<20	<20
		Day 11	<20	<20

Bolded numbers indicate positive ADA ID_50_ titers.

It should be noted that ADA responses in these animals assessed by binding assays, which are not restricted to antigen-binding regions for their inhibitory function, were high following a single infusion of PGT121-YTE with a corresponding reduction in plasma stability and efficacy and included cross-reactive titers against YTE mutants of 3BNC117 and PGDM1400 (Rosenberg et al., 2019). These binding ADA can be demonstrated in macaques lacking neutralization-based functional ADA (Table 1).

### 3.6 Application of the functional ADA assay in a tiered testing strategy for HIV-1 bnAbs

**Fig. 7:**
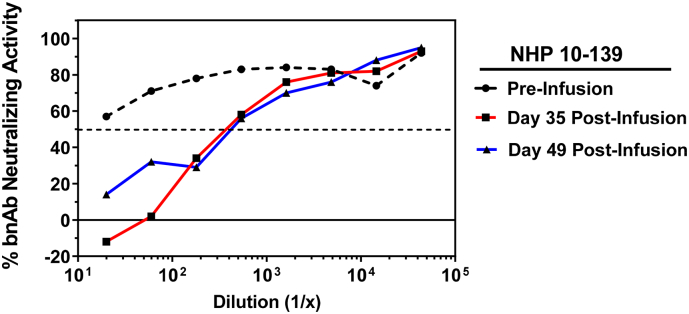
Detection of a functional ADA response against bnAb 10–1074 in NHP. Plasma samples were obtained from monkey 10–139 that received a single passive infusion of bnAb 10–1074 at the following timepoints: day 0 (pre-infusion control) and days 35 and 49 post-infusion. Serial dilutions of plasma were assayed in the presence of 0.016 μg/ml bnAb 10–1074 and HIV-1 Env pseudovirus X2088_c9. The percent neutralization by bnAb in the absence of ADA was 64%.

Where possible, the multi-tiered ADA testing strategy recommends that samples in which ADA activity is detected (Tier 1) and confirmed positive using binding antibody-based assays (Tier 2), be further characterized for functional inhibition in neutralization assays (Tier 3). To this end, we obtained longitudinal plasma samples from NHP that had been passively infused with bnAb 10–1074 alone (*n* = 1), or 10–1074 in combination with 3BNC117 (*n* = 3). These plasma samples were evaluated by a tiered assay strategy using a MesoScale Diagnostics (MSD)-based set of binding assays as reported in the companion manuscript (Bharadwaj et al., 2019). Monkey 10–139 was part of a larger cohort of animals receiving bnAb 10–1074 alone, and was suspected of having generated an ADA response due to a rapid decline in serum bnAb levels by day 35 post-infusion (data not shown). To assess whether this animal may have developed an inhibitory ADA response to 10–1074, we performed the functional ADA assay in TZM-bl cells using the optimized conditions outlined above. Plasma samples from day 0 (pre-infusion control), and days 35 and 49 post infusion were tested using indicator virus X2088_c9 and a fixed concentration of bnAb 10–1074 at 0.016 μg/ml (64% neutralization potency). Anti-idiotype antibodies for 10–1074 and 3BNC117 were utilized as positive and negative controls, respectively (data not shown). While no ADA activity was detected with the pre-infusion control sample (plasma ID_50_ titer <20), robust inhibition of 10–1074 neutralizing activity was observed with plasma samples obtained at days 35 and 49 post-infusion (ADA ID_50_ titers of 378 and 427, respectively) (Fig. 7). These data indicate that monkey 10–139 developed an ADA response following passive infusion of 10–1074, which likely contributed to the rapid clearance of the bnAb from the circulation.

**Fig. 8:**
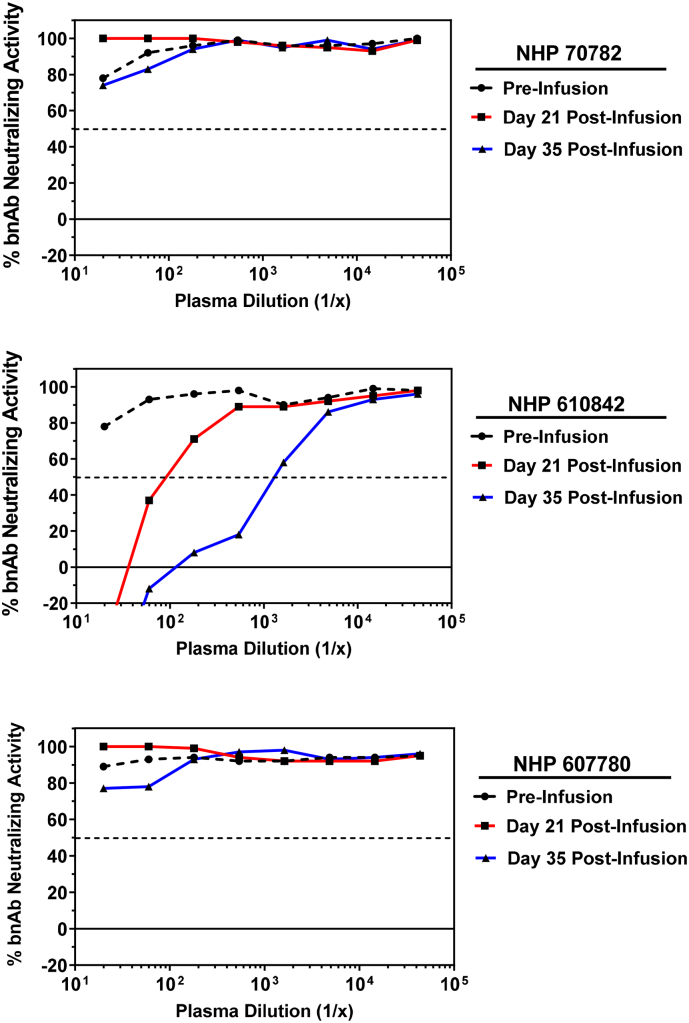
Detection of a functional ADA response against bnAb 3BNC117 in NHP. Plasma samples were obtained from monkeys 70,782, 610,842, and 607,780 that received a single passive infusion of a combination of bnAbs 10–1074 and 3BNC117 at the following timepoints: day 0 (pre-infusion control) and days 21 and 35 post-infusion. Serial dilutions of plasma were assayed in the presence of 0.03 μg/ml bnAb 3BNC117 and HIV-1 Env pseudovirus Q842.d12. The percent neutralization by bnAb in the absence of ADA was 70%.

A second cohort of 3 NHP received co-infusion of bnAbs 10–1074 + 3BNC117. The overall pharmacokinetic measurements demonstrated a more rapid decay of plasma 3BNC117 levels compared to 10–1074, with all animals having no detectable plasma 3BNC117 by day 35 post-infusion (data not shown). To determine if any animals may have developed a functional ADA response to 3BNC117, we performed assays using indicator virus Q842.d12 with an optimized concentration of 3BNC117 bnAb at 0.03 μg/ml (70% neutralization potency). In monkeys 70782 and 607780, post-infusion plasma samples behaved similar to pre-infusion control in which no inhibition of 3BNC117 neutralizing activity was observed, suggesting these animals did not develop a functional ADA response (Fig. 8). In contrast, post-infusion plasma samples from monkey 610842 demonstrated potent inhibition of 3BNC117 activity compared to pre-infusion control (day 21 and 35 post-infusion plasma ADA ID_50_ titers of 90 and 1,302, respectively). These data indicate that monkey 610842 developed an ADA response to bnAb 3BNC117 by day 21 post-infusion that increased in potency by day 35 post-infusion. The positive detection of functional ADA responses in animals 10–139 and 610842, and lack of ADA detection in animals 70782 and 607780, are in agreement with binding assay results using the MSD assay platform (Bharadwaj et al., 2019).

## 4 Discussion

As clinical studies evaluating HIV-1 bnAbs for the prevention and treatment of HIV-1 infection continue to advance, it is critical that qualified assays be developed to detect and quantitate ADA responses that may develop in patients. The FDA recommends a multi-tiered approach for assessing ADA responses that includes assays to measure the functional inhibition of clinical drug product, as the impact of ADA on pharmacokinetics, efficacy, and patient safety may correlate with functional activity rather than ADA incidence (Immunogenicity Testing of Therapeutic Protein Products - Developing and Validating Assays for Anti-Drug Antibody Detection, 2019). In support of this tiered testing strategy, here we describe key experiments that were performed to optimize and qualify an assay for measuring functional ADA against HIV-1 bnAbs.

The TZM-bl assay is a gold standard assay platform that is widely used in the field for measuring HIV-1 neutralizing antibodies, and has undergone rigorous qualification and validation testing to facilitate compliance with GCLP guidelines (Sarzotti-Kelsoe et al., 2014). We have incorporated a simple modification to this assay in which a partial inhibitory dose of bnAb is added to all sample wells, and functional ADA activity is measured as a reduction in bnAb neutralizing activity against a sensitive strain of HIV-1. The key parameter for optimization using this assay format was to determine the optimal concentration range of bnAb to include in the assay. Our results demonstrate that the assay is optimally sensitive and specific when bnAb is present at a dose that results in 40–80% virus neutralization in the absence of ADA. A dose of bnAb that results in <40% virus neutralization was found to result in non-specific activity when the anti-idiotype antibody and bnAb were mismatched. In addition, because the sensitivity of the assay diminishes as the dose of bnAb increases, we recommend 80% virus neutralization by the bnAb as an upper threshold to minimize loss of sensitivity while maintaining a desired level of ADA detection.

It was evident from these bnAb dose optimization experiments that free drug interferes with the sensitivity to detect functional ADA in a dose-dependent manner. The level of interference by residual bnAb present in a patient serum sample will depend on the specific bnAb and virus utilized for functional ADA measurement. Furthermore, the level of interference in the ability to measure a patient-derived ADA response might not be accurately predicted by the use of mouse anti-idiotype control antibodies. These results highlight the importance of measuring ADA in clinical trial samples at kinetic timepoints in which the serum levels of passively infused bnAb have declined to concentrations that do not exhibit detectable neutralizing activity against the ADA target virus in the standard TZM-bl neutralization assay. Given that new and improved variants of bnAbs are being engineered with modifications to dramatically increase in vivo half-life (Ko et al., 2014), the ability to measure potential development of functional ADA in these patients at desirable time points becomes more restricted. A method that uses PEG precipitation and acid dissociation to overcome drug interference in ADA assays has been previously described (Zoghbi et al., 2015). The compatibility of this approach with the functional HIV-1 bnAb ADA assay described here warrants investigation.

Using a panel of four different murine anti-idiotype antibodies, we observed high levels of sensitivity in detecting bnAb inhibition under optimal assay conditions. LOD ranged from IC_50_ titers of 0.31–5.0 μg/ml. The lower level of sensitivity observed with the anti-PGT121antibody likely reflects the lower potency of this anti-idiotype mAb rather than a lack of sensitivity of the assay, as poorer sensitivity of this anti-PGT121 antibody was also observed in the binding antibody ADA assay formats (Bharadwaj et al., 2019). The linearity of the ADA neutralization curves also varied depending on the anti-idiotype antibody and the dose of bnAb used. The neutralization reduction curve for a potent ADA was generally linear between 30% and 75% neutralization values, and proportional to the concentration of ADA mAb in the sample.

A panel of five HIV-1 bnAbs currently in clinical development and their respective murine anti-idiotype antibodies were used to demonstrate a high degree of specificity in detecting matched functional ADA responses under optimal assay conditions. The specificity and sensitivity of ADA activity was further observed to be similar whether anti-idiotype mAbs were diluted in PBS or spiked into normal human serum samples, which is the natural matrix when testing clinical patient samples. Lastly, a high level of assay precision was demonstrated by successful intra- and inter-operator repeatability measurements. Using 3BNC117 and 10–1074 bnAbs with their matched respective anti-idiotype antibodies, we observed that 100% of ADA IC_50_ titers were within 2-fold of the mean, and %CV for all intra- and inter-operator runs was 25% and 43%, respectively.

While the optimized conditions for measuring anti-HIV bnAb ADA were determined using murine anti-idiotype antibodies as positive controls, we demonstrate that this assay performed with high sensitivity and precision for measuring naturally occurring ADA responses that developed in rhesus macaques following passive infusion of HIV-1 bnAbs. Importantly, using a set of NHP plasma samples that were also analyzed using the tiered approach with an MSD binding-antibody ADA platform, we demonstrated concordance in identifying animals that were both positive and negative for anti-bnAb activity. These data highlight the utility of using the functional anti-HIV bnAb ADA assay described here in conjunction with the optimized MSD platform in a multi-tiered approach for detecting, quantitating and characterizing anti-HIV bnAb responses in clinical trial participants.

In addition to functional ADA that inhibits neutralization of bnAbs, ADA can be monitored in binding assays to detect a broader population of ADAs that are not limited to specific interactions with the antigen contact residues. Functional ADA can reach high levels in unprimed macaques following multiple injections, or after a single infusion into animals with pre-existing ADA (Rosenberg et al., 2015). By contrast, ADA responses detected using binding assays are observed at high titers following both single and multiple injection/s and impact both pharmacokinetics and efficacy of protection in the absence of neutralization-inhibitory ADA, which appear to require affinity maturation to exhibit optimal potency.

## 5 Conclusion

The results demonstrate the utility of the qualified assay to detect functional ADA as a measure of reduction in bnAb neutralization potency. The assay format described here can be easily adapted for measuring functional ADA for other HIV-1 bnAbs of interest. However, it is recommended that additional bnAb-specific assay qualification assays are performed to identify optimal conditions. Thus, a known positive control anti-idiotype antibody should be assayed in the presence of at least three different concentrations of bnAb (e.g., concentrations that produce approximately 40%, 50%, and 80% neutralization). The results will help establish the impact of drug interference and can be used to determine the expected titers of the positive control under optimal assay conditions (~50% neutralization by bnAb, range 40–80%). Our data demonstrate that 30% inhibition of bnAb neutralizing activity can be used as a statistical cut-point for defining positive functional ADA activity, however we recommend the use of 50% inhibition (i.e. serum ID_50_ titer) when defining ADA responses given that 50% inhibition is typically on the linear portion of the ADA titration curve and thus is a more stringent and less variable measurement. When performing the ADA assay using clinical trial specimens, we recommend the following criteria for acceptable assay performance: i) the concentration of bnAb results in 40–80% neutralization in the absence of ADA (i.e., optimal balance assay sensitivity and specificity), ii) average RLU in virus control wells (without bnAb) are >10 times higher than average RLU in cell control wells (without virus), and iii) positive control anti-bnAb exhibits expected activity. It is also recommended that a pre-infusion baseline control sample be tested in parallel with post-infusion timepoint samples to account for any pre-existing inhibitory activity in the patient's serum.
